# Freeze-Chilling of Whitefish: Effects of Capture, On-Board Processing, Freezing, Frozen Storage, Thawing, and Subsequent Chilled Storage—A Review

**DOI:** 10.3390/foods10112661

**Published:** 2021-11-02

**Authors:** Ulf Erikson, Solveig Uglem, Kirsti Greiff

**Affiliations:** 1 Department of Aquaculture, SINTEF Ocean, 7465 Trondheim, Norway; 2 Department of Fisheries and New Biomarine Industry, SINTEF Ocean, 7465 Trondheim, Norway; Solveig.Uglem@sintef.no (S.U.); Kirsti.Greiff@sintef.no (K.G.)

**Keywords:** whitefish, freeze-chilling, value chain, processing, product quality

## Abstract

The current review investigates how whitefish quality is affected by capture at sea, on board handling, freezing, double freezing, frozen storage, thawing, and chilled storage. Packaging of fillets in MAP and vacuum are also covered. The main goal was to evaluate the freeze-chilling concept as a possible method for the fishing industry for all-year-round marketing of fish captured during the relatively short fishing period. The review covers both the effect of each processing step in the supply chain as well as the combined effect of all steps in the chain from sea to consumer, including post-thawing chilled storage, defined as the freeze-chilling method.

## 1. Introduction

**Figure 1:**
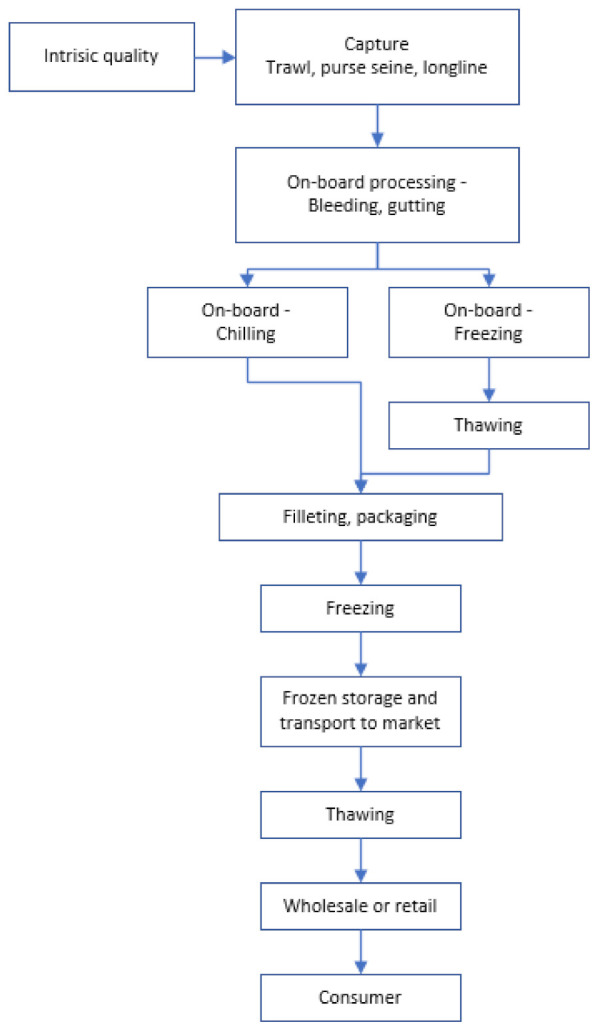
The freeze-chilling value chain for whitefish showing the involved steps from sea to consumer. Frozen storage may take place at plant before transport to market for off-season marketing or continued near market until thawing, that is, consumer packages can be made at plant or near market. The current review deals with the following important factors that collectively determine fish quality as experienced by consumers: intrinsic quality, fishing gear damages, capture stress, bleed-out, rigor mortis status at the point of freezing, and subsequent temperature–time history during freezing, frozen storage, thawing, and ultimately, chilled storage.

The concept of freeze-chilling is commonly defined as freezing of food products (here whitefish) before thawing and chilled storage in market (fishmongers and supermarkets). The concept can represent logistic benefits in product distribution and retailing as well as enabling the fish industry to supply thawed fish to the market off-season. For whitefish, typical products would be whole fillets, or fillet pieces, packed in vacuum or modified atmosphere (MA). For marketing of high-quality products, it is rather obvious that each processing step requires special attention to achieve the best possible quality and sufficient shelf life once the product is thawed. Sub-optimal processing conditions may lead to significant quality degradation of the products. In case of whitefish from the North Atlantic, a typical freeze-chilling supply chain would comprise the processing/operations shown in Figure 1.

Some fishing companies in Norway are currently considering focusing more on freeze-chilling, which would imply that the fillets or related products (e.g., fillets packed in vacuum or MA) are either refrozen at the plant after thawing (double freezing) for later supply to market, or alternatively, transported in the frozen state for thawing, processing, and packaging near the market. The main fishing season for North Atlantic cod (*Gadus morhua*) lasts for about four months, from mid-January to mid-April. This means that the duration of the frozen storage period can be up to about eight months if continuous off-season marketing is considered.

The scope of the current review is to assess what effects of the various steps in the value chain may have on fish quality, both considered individually (Chapter 2) and collectively (Chapter 3, freeze-chilling). Only lean whitefish are considered in the present review and it is not our intention to describe chemical and microbial changes occurring in fish in detail. Nor are different freezing and thawing technologies covered herein. Overviews of relevant technologies are: freezing and frozen storage [1,2,3,4], thawing technologies [5,6,7], processing and packaging [8], and seafood cold chain [9].

## 2. Effects of Individual Processing Steps in the Value Chain

### 2.1. Intrinsic Quality and Effects of Capture

The intrinsic quality of whitefish is determined by fish size, season of catch, sexual maturation, fishing ground [10,11,12,13], and catching method [14,15,16]. Margeirsson et al. [17] conducted a survey of how fillet yield, gaping, number of nematodes, and prevalence of bruises of trawl-caught cod was affected by capture conditions. The study comprised data from several Icelandic vessels and processing plants over a period of 29 months. It was found that fishing ground had a significant effect on fillet yield, gaping, and number of parasites, and that time of year had a significant effect on all variables. Moreover, the time-lag from catch to processing had a significant effect on gaping and bruises, whereas fillet yield correlated with condition factor and head proportion.

Effects of catch size and onboard storage of cod caught by otter trawl was studied by Botta and Bonnell [18]. The initial quality of fish from hauls ≥ 9.5 tons was considered quite good, although not as good as fish from hauls ≤ 2.5 tons as the latter group of fish achieved a significantly higher quality grade. Generally, significantly higher quality was obtained when catch sizes were reduced to <5 tons.

Recently, several studies have been conducted to improve fishing gear to obtain higher product quality of the catch. Quality in this context refers to various capture damages, which would also mean an economic loss due to downgrading in the market. Fishing gear damages do not only affect the external appearance of fish, but also factors like cuts deep into the muscle, squeezing fish together, which in turn may cause pressure damages, internal haemorrhages and discoloration of flesh. Veldhuizen et al. [19] provide an overview of typical injuries and mortality when fish are caught by common types of fishing gear. Their review showed that greater capture depth and longer fishing duration were associated with more injuries and higher mortality. Large changes in water temperature, long duration of air exposure, and high density in the net were associated with higher mortality. In an attempt to reduce the extent of external damages (gear marks, pressure injuries, skin discoloration, bruises and abrasion) to cod caught by trawl, Tveit et al. [20] compared fish caught by the traditional gear with a two-panel knotted codend with a four-panel knotless codend. Overall, however, no significant differences in the extent of injuries were found. Digre et al. [21] conducted a study on a trawler where cod and haddock (*Melanogrammus aeglefinus*) were simultaneously captured by using two trawls in a double rig fitted with traditional and ‘T90′ codends. In terms of quality-related parameters (gear injuries, scale loss, pressure injuries, bruises, pH, rigor mortis, fillet color, blood spots, and gaping), haddock caught with traditional trawl net had more external injuries than their ‘T90’ counterparts. No other differences in fish quality were observed.

In contrast, cod quality was improved when a new dual sequential codend was used. Compared with the traditional trawl gear, the extent of catch damages (poor exsanguination, gear marks as well as skin discoloration, bruises, and abrasion) were significantly reduced [22]. The extent of external damages of haddock captured by demersal trawl with the compulsory sorting grid and diamond mesh codend gear configuration was studied by Sistiaga et al. [23]. Capture of haddock without inflicting gear damages (gear marks, ecchymosis, bleed-out, skin abrasion and pressure) was considered challenging although the severity of most damages was low. Removal of the grid and changing the design of the codend (“gentle codend”) can significantly reduce the extent of damages. Further tests with cod confirmed that by substituting the compulsory gear with a four-panel selective knotless section followed by a gentle codend increased the probability of catching cod without damages. In addition, a significant reduction in the severity of all catch damage categories (gear marks, ecchymosis, bleed-out, scale loss) was observed [24].

Some new ideas to improve fishing practices have in fact been shown to have detrimental effects on fish quality. To facilitate continuous processing of the catch on board, the effect of the so-called buffer towing was assessed by Brinkhof et al. [25]. The concept embraces the idea that when the desired amount of fish is caught, or exceeded, before the previous haul is processed, the trawl is lifted off the seabed and towed slowly at a given depth until the production capacity on board is restored. However, the results showed that buffer towing has significant negative impact on fish quality in terms of poor exsanguination, increased fillet redness, gear marks, and discoloration, bruises, and abrasion of skin. Furthermore, de Haan et al. [26] assessed whether electrical pulse trawl fishing of cod could replace trawl fishing using mechanical stimulation. Electrical stimulation resulted in haemorrhages and various injuries including spinal fractures to certain fractions of the fish. The probability for injuries increased with field strength and to some extent with duty cycle, while it decreased with pulse increasing frequency (100 to 180 Hz). For market-sized cod, the injury probability tended to decrease with body size.

The capture methods used for whitefish generally imply that the fish are subjected to stress [27,28]. Hence, it can be assumed that phosphocreatine, ATP, and glycogen stores are more or less depleted once the fish are taken aboard. It is well-known that handling (capture) stress can affect flesh quality. It should be pointed out, however, that this effect of stress is less severe in case of sedate species such as cod compared with active swimmers such as Atlantic salmon (*Salmo salar*) [29,30]. Capture stress can be quantified by measuring the initial pH in white muscle just after fish are taken on board and killed. The pH values reflect the degree of struggling and escape swimming during the fishing operation. Preferably, the measured values should be put in context by comparison with pH values of rested and exhausted fish of the same species (if such data are available). Initial pH values in the white muscle of trawl-captured cod have been reported as pH 6.97 ± 0.16 [28] and pH 7.16 ± 0.07 [31]. Other reported initial values are pH 7.11 ± 0.03 (cod) and pH 6.93 ± 0.93 (haddock) with corresponding times to rigor onset < 8.0 h and 9.5 h, respectively [32], and pH 7.2–7.3 ± 0.0 (cod) and pH 6.7–6.9 ± 0.0 (haddock), where time to rigor onset for both species was about five hours [21]. By comparison, the pH in rested white muscle of cod is 7.6 ± 0.0 and the corresponding time to rigor onset is then 18–20 h post mortem for fish stored in ice [30].

### 2.2. On Board Processing

On trawlers, after the captured fish are hauled on board, they are commonly transferred to a dry bin (a steel tank without water located under the shelter deck) where the fish gradually die unless they are processed shortly after capture. In practice, this means that live, moribund, or dead fish are subsequently gutted, bled, washed, and frozen (or stored in ice for delivery as fresh fish). In case of large catch sizes, delayed processing implies that a large portion of the catch dies before processing is completed. The implication of delayed bleeding is that the blood coagulates within the fish and will therefore be difficult to remove. Residual blood in fillets, due to poor bleed-out, can result in discolorations [14,28,31]. Fillet whiteness is regarded as an important quality trait in whitefish. Therefore, the amount of residual blood in fillets should be minimized. Hence, adequate bleed-out is required. Bleeding method has been shown to be of less importance, the important factor is that fish should be bled before the blood starts to coagulate. Indeed, Olsen et al. [33] reported that cod should be bled immediately after capture no later than 30 min, or no later than 60 min post mortem [18]. The effect of bleed-out time (5 vs. 30 min) and bleeding medium (seawater vs. slurry ice) on the quality of cod caught by trawl was studied by Nguyen et al. [34]. Bleeding time had merely a minor effect on sensory, microbiological, and biochemical quality of cod fillets. However, bleeding in seawater resulted in more rapid microbial and biochemical developments. The results suggest that a short bleeding time (5 min) is sufficient for proper drainage of blood. Correspondingly, it can be mentioned that when cod are bled in air by gill cut immediately after killing, it was observed that the flow of blood from the gills basically ceases after one to two minutes [27].

Subsequently, the fish should be processed and frozen without delay to minimize autolysis and to obtain the advantageous effects on quality by freezing while the fish are in the pre-rigor state. Considering time to rigor onset (see above), pre-rigor freezing is possible [31], at least when fish are processed without delay in cases where the catch sizes are small to moderate. It is well established that for the best possible product quality, fish should be frozen before the onset of rigor mortis [10,15]. Tobiassen et al. [35] concluded that pre-rigor processing is a better concept for distribution and sale of fresh cod compared with in-rigor and post-rigor processing. Pre-rigor fillets had less gaping, firmer texture and slower bacterial growth after filleting. However, the shelf life of pre rigor fillets (wrapped in plastic film during ice storage) was reduced compared with the two other treatments, probably since in- and post-rigor fish were filleted at later stage and were therefore exposed to atmosphere for a shorter period of time.

In practice, however, fishermen tend to delay catch handling since they prefer to wait until the captured fish have become calm, moribund, or dead. Also, when catch sizes are large, processing can take several hours which means that the processed fish can be frozen in various rigor states. To enable immediate, easy and safe (EHS) processing of the catch, wriggling fish can be immobilized by electrical stunning just after capture [36]. The concept has been introduced on several vessels to facilitate rapid catch processing to reduce discolorations due to delayed bleed-out. Another benefit of using the method would be to extend the pre-rigor period which means that a larger proportion of the fish can be frozen in the pre-rigor state. For large catch sizes though, electrical immobilization might still not be sufficient to enable processing of live fish. Therefore, electrical immobilization can be combined with the use of live storage tanks on board from where the fish are consecutively killed and immediately processed [36]. Short-term live storage of whitefish on board has been studied by Olsen et al. [28], Digre et al. [32], and Erikson et al. [31].

In the case of when fresh fish on ice is going to be landed, ice storage in pens is preferable to ice storage in boxes. When iced fish were landed later than six days post mortem, quality was considered low. The reduced quality at sea was primarily caused by bruises and discoloration. In addition, poor texture upon landing was another issue caused by chilled storage [18].

### 2.3. Freezing

Considering the current value chain, freezing takes place on board vessels and after landing at the processing plant, either as freezing on board of fresh fish, or more commonly, after thawing and processing (double freezing) before transport to market. Freezing on board typically takes place in vertical or horizontal plate freezers at −30 to −40 °C. At sea, a typical example would be freezing of bled and gutted cod in 100 mm blocks, with an initial fish temperature of 5 °C. The fish will then be frozen after 3 h 20 min when a vertical plate freezer operating at −40 °C is employed [1]. Under such conditions, the center of a block can be reduced from 0 to −15 °C in about three hours [10].

As discussed by Hedges [2], the effect of freezing rate in fish has been poorly studied. When slow vs. rapid freezing is compared in terms of fish quality, there seems to be no clear consensus as both advantages and no advantages of rapid freezing are reported. These observations may be explained by various factors such as initial benefits of rapid freezing can be offset by subsequent frozen storage or thawing. Lean fish, like cod and haddock, contain 75–80% water where freezing begins between −1 to −3 °C. Most of it freezes between −1 and −5 °C (the critical freezing zone) and at −25 °C, 90 to 95% of the water is present in the frozen state [4]. For example, when the temperature is lowered from −1 to −20 °C, the amount of unfrozen water in cod fillets drops from 92 to 11% [2]. It is generally accepted that the freezing rate should preferably be rapid. Common freezing rates are about 1 to 2 cm h^−1^, which has been found to be adequate to reduce unwanted changes in quality [37]. Freezing time can be reduced by increasing the air speed around the product although air speeds above 5 m s^−1^ seem to have little effect. When fish packages are frozen, freezing time might be longer, particularly if packages contain trapped air [1]. Regarding the effect of freezing temperature on tissue damages, Anderssen et al. [38] showed by using MRI that tissue damages in vacuum-packed postrigor cod loins after thawing (4 °C circulating water for 2 h) were minimized by using blast freezing at −40 °C with circulating air at 3 m s^−1^ compared with at −20 and −5 °C, both in stagnant air. The liquid loss after freezing and thawing at −5, −20, and −40 °C diminished with decreasing temperature as 6.0, 3.9, and 1.7%, respectively.

The effect of freezing method on cod quality was studied by Erikson et al. [39]. Rested cod, with aerobic tissues (to minimize in vivo glycolysis) were frozen immediately after they were killed, gutted, washed, wrapped in plastic sheets, and put into cardboard boxes. The fish were frozen in magnetic field (MF), air-blast (AB), and cold storage (CS) freezers. In the MF, fish were initially frozen at −45 °C for 16.5 h under the influence of a static and pulsating magnetic field, before the temperature was lowered to −30 °C for further storage. In AB, freezing took place at −35 °C at an air speed of 5 ms^−1^. After 15 h, the boxes were moved to the CS freezer. Some boxes were put directly in CS, where the temperature was −27 °C without forced convection of air. All fish were stored for 46 days before thawing in air at 3.4 °C. Quality analyses were carried out 47 h after start of thawing. Core temperature measurements showed that the mean durations the fish stayed in the critical temperature zone (−0.8 to −5.0 °C) were 67, 98, and 478 min for MF, AB, and CS, respectively. Despite the much faster freezing rates for MF and AB compared with CS, largely no significant differences were observed in terms of fillet microstructure, area fraction distribution of muscle fiber tissue, connective tissue, interstitial spaces, and mean thicknesses of open spaces between white muscle fibers. Furthermore, no significant differences between freezing methods were observed in terms of external appearance of gutted fish after thawing and, indeed, fillet quality as defined by ultimate pH, water content, drip loss, water holding capacity (WHC), NMR T_21_, and T_22_ relaxation times and A_21_ and A_22_ populations, ATP, IMP, Hx, K-values, gaping, and hardness. Overall, the thawed fish were regarded as a high-quality product. After further ice storage for six days, no significant effects of freezing method were observed (only deterioration due to ice storage per se). Thus, the considerable slower freezing rate in case of CS did not affect quality.

This finding can be explained by that the freezing rate is of almost no importance when fish are frozen pre-rigor due to the fact that most of the water is still located inside the cells where the ice crystals are formed. However, freezing times longer than six hours should be avoided, and in the case of very slow freezing rates, in the order of 20 h, very large ice crystals can be formed, which will result in inferior quality such as poor texture, high drip loss, and accelerated rancidity [3]. The reason for these changes is that at rigor onset, pH has been reduced causing myofibrils to shrink. The intracellular fluid is expelled and accumulates in extracellular spaces, especially between fiber bundles, which constitutes a likely source of any drip [40].

Connell and Howgate [41] studied the effect of freezing and thawing of cod without intermediate frozen storage. If carried out rapidly enough, the effect of freezing and thawing were without effect on freshness characteristics. On the other hand, firmness and dryness did increase, and when thawing was carried out slowly overnight, some freshness was lost if very fresh fish are initially frozen. It was concluded that textural changes due to a single cycle of freezing and thawing could be easily detected by a trained sensory panel. Color, on the other hand, was not affected.

The impact of freezing temperature (−10, −25, −40, −55, and −70 °C) for 10–11 days on farmed cod fillets packed in polyethylene bags was studied by Mørkøre et al. [42]. The fish were filleted, packed, and frozen four days post mortem. After slow thawing at 2 °C, when the core temperature of the fillets reached 0 °C, appearance, gaping, liquid leakage, texture, and thaw exudates were determined. A complex impact of freezing on quality was reported. Freezing at −10 and −25 °C increased thaw exudates and weight loss, particularly at the highest temperature. However, fillets frozen at −10 °C had the whitest appearance and lowest occurrence of gaping. Regarding toughening during storage, fillets exposed to −10 and −55 °C seemed to be more affected than at all other temperatures. After three days of chilled storage, the weight loss at all temperatures varied between 9 and 12.5%, whereas fresh fillets had a weight loss of 5.5%. Overall, no beneficial effects were found by decreasing temperature below −40 °C.

### 2.4. Frozen Storage

Various chemical reactions affecting product quality during frozen storage have been described by Haard [43] and the effect that frozen storage has on muscle proteins is treated by Mackie [44]. It is well known that during frozen storage, fish undergo changes in flavor, odor, texture, and color. While bacterial growth during frozen storage is largely inhibited, it is important to realize that dormant bacteria can grow after thawing during subsequent chilled storage. A temperature goal of −18 °C is recommended during frozen storage [45,46]. Long-term frozen storage can, however, reduce or alter bacterial composition during chilled storage. It is generally accepted that if high-quality fish are frozen reasonably rapidly and the storage period is relatively short, changes in sensory quality after thawing and cooking are quite small. For longer storage times, quality tends to gradually deteriorate. Furthermore, temperature fluctuations can also increase the rate of quality degradation during storage. It has been recommended for lean demersal fish that the storage temperature should be between −24 to −30 °C (see Hedges [2]). Especially, Gadidae species are vulnerable to toughening during frozen storage due to considerable denaturation and dehydration. Several factors are involved in the mechanism behind this phenomenon (see LeBlanc et al. [47]). With higher frozen storage temperature (−7, −10, or −18 °C), the rate of textural deterioration increases due to protein denaturation. After frozen storage, the toughness of cod muscle increases accordingly, whereas the cohesiveness decreases. In turn, these changes are related to the loss of WHC [48]. The effect of frozen storage of cod fillets, for up to 62 weeks at −20 and −30 °C, on protein aggregation and related changes in texture and functionality was studied by Careche et al. [49]. The time dependent formation of aggregates, consisting of largely myosin and actin, correlated with shear resistance, which was more prominent at −20 °C than at −30 °C. The effect of frozen storage for up to 30 weeks at −10 and −30 °C on cod and haddock texture was assessed by Badii and Howell [50]. The fish were frozen 48 h post capture and fillets, wrapped in polythene, were thawed for 4 h at 20 °C. For both species, the rate of protein denaturation and muscle toughening was highest at −10 °C, as demonstrated by much higher compression forces increasing considerably from 18 to 30 weeks of frozen storage. Consequently, fillet water contents after 30 weeks were about 5.4% lower at −10 °C than at −30 °C for both species. On the other hand, an advantage of freezing fish before the onset of rigor mortis was demonstrated by MacCallum et al. [10], where cod fillets were frozen pre-rigor and stored at −23 °C. In this case, the toughening reaction elapsed very slowly. Under such conditions, overall texture scores were maintained for six months.

The effect of frozen storage temperature and storage time on cod quality was also studied by Burgaard and Jørgensen [51]. Fish caught by trawl were filleted after three days on ice (post rigor) where loins were vacuum-packed before freezing overnight in a blast freezer at −40 °C. Storage temperatures ranged from −10 to −80 °C in ten-degree intervals and the fish were stored from 1 up to 18 mo. Depending on type of analysis, the fish were thawed at 2 °C overnight or in water at 8 °C. The following indices of quality were assessed: drip loss, WHC, LF NMR relaxation times, color, thiobarbituric acid reactive substances, and sarcoplasmic reticulum Ca^2+^-ATPase and lysosomal Cathepsin D activities. Notably, storage up to 12 mo did not show significant differences between −30 °C and lower temperatures. For longer storage times, only water distribution (NMR measurements) varied to some extent when storage at −30 °C was compared with −40 °C or lower. Regarding color, loins stored −10 and −20 °C were significantly lighter and more yellowish than loins stored at lower temperatures.

Headed and gutted Pacific whiting (*Merluccius productus*) stored for 0, 2, and 5 d at <4 °C were subsequently filleted, frozen at −18 and −80 °C, and analyzed after 24 weeks. TMAO (trimethylamine-N-oxide) activity decreased more quickly at −18 °C, inducing a gradual increase in formaldehyde (FA) up to 24 weeks, while FA was near zero after 12 weeks at −80 °C. Formation of FA lead to protein denaturation and textural toughening (protein aggregation), low water retention and low salt soluble protein solubility [52]. Gutted Mediterranean hake (*Merluccius merluccius*) were acquired from the local market on the day of capture. Fresh and frozen-thawed fish were monitored during ice storage for up to 14 days. The frozen-thawed fish, wrapped in plastic foil and kitchen foil, had been stored at −20 °C for six months before thawing overnight at 4 °C. The authors concluded that in terms of sensory marks, spoilage of frozen/thawed hake occurred at an earlier stage during ice storage compared with fresh hake. On the other hand, when chemical and microbiological parameters were considered, changes occurred much later in frozen/thawed fish [53]. In line with this, it has been reported that when cod are subjected to frozen storage for more than five weeks, the bacterial numbers are reduced [54,55]. Changes in lipids in cod and haddock during frozen storage for up to one year at −10 and −30 °C were studied by Aubourg and Medina [56]. For both species, most lipid damage indices showed significant correlations with storage time at −30 °C with little difference between species. At −10 °C, however, haddock exhibited higher lipid oxidation (peroxide value and TBA index) and hydrolysis (FFA content) than cod did.

The color characteristics of cod fillets has been compared just after bleed-out on board a trawler vs. after frozen storage for 61 days. Beheaded and gutted fish were frozen in a vertical plate freezer reaching a block temperature of about −20 °C after 3.5 h. Blocks were packed in woven polypropylene/paper bags and stored on board at −23 °C for three days and subsequently at −24 ± 4 °C after the fish were brought ashore. The fillet color characteristics of cod from various treatments on board and after frozen storage followed a similar pattern. However, compared with fresh fillets, frozen/thawed fillets exhibited a yellowish tint (CIE b*, hue and color saturation) after frozen storage regardless of on-board processing method. Fish subjected to five-hour-delayed processing (unbled) resulted in fillets with inferior color characteristics [31]. By comparison, both cod and haddock fillets became yellowish at −10 °C for 65 weeks, but at −30 °C, the color remained white [57].

Whitefish frozen on board vessels just after capture is generally regarded as a high-quality product, and therefore, it could be conceivable to make various fish products on board. Bøknæs et al. [58] conducted a study with cod caught by freezer trawler (tow duration 4 h, catch size 6 tons) where the catch was processed from a single haul as five different batches: (A)—glazed portions, (B)—glazed vacuum-packed portions, (C)—unglazed portions, (D)—interleaved packed fillets, and (E)—double frozen fish. The fish were bled in seawater (30 min at 4 °C) before they were headed and gutted (HG) pre-rigor. Subsequently, the fish were machine filleted and skinned. Whole HG fish were frozen in horizontal plate freezer and packed in cardboard boxes, whereas fillets from batch D, and fillets from batch A, B, and C cut by hand into loin and tail portions were individually quick-frozen (IQF) in a blast freezer until the core temperature reached −28 °C after 70 min before packing in PE-bags and cardboard boxes. Fish from all five batches were placed in cold storage on board and subsequently, fish from all treatments were subjected to frozen storage for 13, 26, 35, and 46 weeks at −28.0 ± 3.2 °C. HG cod (E) were thawed industrially with an initial water temperature of 30 °C equalized overnight to 0 °C before machine filleting and re-freezing (double frozen) after a few hours. Batch (B) cod portions were vacuum packed. Frozen fillets and portions were packed in PE-bags and thawed overnight in air at 5 °C before analysis. Product quality was assessed as WHC, water loss on cooking, and sensory quality indices of cooked samples. The glazed portions, vacuum-packed portions, and interleaved packed fillets were all considered as high-quality products after 46 weeks frozen storage. In contrast, unglazed portions and double frozen fillets had developed cold storage odor and flavor as well as very dry fibrous texture during frozen storage, indicating that glazing effectively protects against dehydration. It should be mentioned though that glazing constituted 5–7% of portion weight. After 26 weeks, freezer burn appeared in unglazed portions causing a poor appearance. Compared with all other treatments, the highest water loss and lowest WHC were clearly evident in the case of double frozen fillets. The authors concluded that the quality of cod can be greatly improved by altered processing compared with the more common double frozen cod products. The recommended practices to produce high-quality products were short tow duration, small catch size, rapid gutting, and bleeding less than six hours between catching and freezing as well as storage below −20 °C. Furthermore, it was found that glazing effectively protected against dehydration. Regarding marketing aspects, the authors concluded that high-quality products can be produced at sea and be suitable for retail and catering markets.

### 2.5. Thawing

The temperature and duration of the frozen storage period should be taken into consideration before thawing. When fish are frozen pre rigor, ATP and glycogen in white muscle will be present at some level, although partial degradation to IMP and lactate, respectively, have already occurred as a result of capture stress and possibly handling on board. Furthermore, the freezing process per se may lead to further degradation. For instance, during blast freezing, ATP and glycogen contents have been reduced by 28 and 59%, respectively [59]. Depending on storage temperature and time, ATP can still be present when the fish are thawed, meaning there is a risk that thaw rigor (very strong rigor causing loss of tissue water, dry texture, and gaping) can occur. In cod, it has been shown that ATP is stable for at least 12 weeks at −40 °C and at −20 °C the ATP will eventually be degraded. By using proper thawing conditions, thaw rigor can be avoided by either keeping the fish in a frozen state until ATP is degraded or by slow thawing (low temperature) [60]. Stroud [61] recommended eight week storage at −29 °C to avoid thaw-rigor by letting the fish slowly pass through rigor in the frozen state. On the other hand, Hurling and McArthur [62] reported that rapid thawing of cod is preferable to slow thawing when fish are to be refrozen to avoid greyer appearance and staler taste after cooking. Slow thawing may occur during processing in plants when blocks are large and heat transfer is sub-optimal. Cai et al. [7] also concluded, based on a review, that thawing rates should be quick to minimize drip loss, which results in dry, stringy texture, and less tasty fish. Another thing to pay attention to is that the target temperature, after the thawing process is complete, should be low to avoid accelerated enzymatic and microbial reactions. Notably, a variety of thawing methods have been tested for seafood and most of them have shown desirable effects of thawing once the process parameters are optimized.

The concept of thawing sea-frozen fish in water was studied by Hewitt [63]. Cod caught by a trawler were gutted, headed, and frozen on board in blocks at −40 °C within one hour. At the laboratory, thawing took place as humid air-blast thawing or in water at 15 to 21 °C to compare quality and weight gain. Fish quality and fillet yield were similar and considered good for both thawing methods. Fish thawed in water gained 1–2% in weight, whereas no changes in weight took place in case of thawing in air. During subsequent chilled storage, the corresponding weight losses after 48 h were 4.5 and 2.5%, respectively. Results from a taste panel showed no significant quality differences between the two thawing methods. However, fillets thawed at 21 °C exhibited more gaping and were softer than fillets thawed at lower temperatures. A maximum water temperature of 18 °C was therefore recommended. Genc et al. [64] studied quality in terms of color, pH, TVB-N, texture, and bacterial counts of meagre (*Argyrosomus regius*) fillets subjected to different thawing methods. Filleting took place about six hours post mortem before individual freezing in blast and fluid bed freezers at −20 °C. Fillets were then glazed by immersion in water at 0.2–0.4 °C for 30 sec before frozen storage for 21 days at −20 °C to simulate typical retail conditions. Thawing took place in a refrigerator (4 °C for 6 h), in air (16 °C for 5 min in closed polyethylene bags), or in a microwave oven (15 min at 90 W). Overall, the slow refrigerator thawing was found to be the preferred thawing method. From a microbiological perspective though, Martinsdóttir and Magnússon [15] recommended thawing sea-frozen fillets at low temperatures such as at 0–1 °C (which would imply slow thawing) to avoid possible growth of unwanted surface bacteria.

### 2.6. Double Freezing

Double freezing is in the present context relevant when fish frozen at sea are thawed at processing plant before processing and re-freezing, as fillet products transported to the market (Figure 1). The effect of double freezing of trap-caught Atlantic cod was studied by MacCallum et al. [10]. Unfrozen, once- and twice-frozen fish were assessed by organoleptic and chemical methods after storage at −23 °C. Furthermore, effects of pre- and in-rigor processing as well as catching season were also taken into account. The sensory panel showed a clear preference for fish frozen in the pre-rigor state, followed by in-rigor frozen fish and then by pre-rigor double frozen fish. Very good quality could be obtained for double frozen cod by holding gutted fish at −46 °C before thawing (blocks in recirculating tap water at 18 °C) and refreezing and storage at −23 °C. Thaw-drip, however, was less in once-frozen fish. Hurling and McArthur [62] studied the effect of thawing and refreezing of cod fillet blocks on muscle functionality and sensory attributes. Compared with once-frozen fish, refreezing did not necessarily cause greater deterioration of cooked fish sensory attributes after nine months at −22 °C. However, refreezing induced faster decline in protein solubility and reduced WHC, although NMR proton relaxation times indicated no relocation of tissue water. Compared with short thawing time (tap water at 18 °C for 45 min), long thawing times (in air at 5 °C for 30 h) before refreezing and storage, resulted in greyer appearance and staler flavor of cooked fish. Notably, the observed changes in muscle functionality were not directly correlated to sensory attributes. Kristinsson et al. [65] compared the effect of single and double freezing at −20 °C of red hake (*Urophycis chuss*). The experimental design included two different holding times, 6 and 24 h of thawed fish between the first and second freezing. After three months, once-frozen fish had the lowest amounts of dimethylamine (DMA), whereas double-frozen fish, with 24 h holding time, had the highest amounts. After seven months, however, no significant differences between treatments were observed. Protein solubility was reduced by 50% in all treatments. The effect of double freezing on cod fillet quality was studied by Schubring [66]. HG fish in different states of rigor were frozen on board (−35 to −40 °C) a fishing vessel, stored for ten days (−30 °C), thawed (immersion in tap water at 16.5 °C for 15 h), and filleted before refreezing (−35 to −40 °C) and storage (−24 °C) for six weeks as breaded and battered portions. Texture (gumminess, and to some extent firmness) was different, irrespective of rigor state, when single and double freezing were compared. The latter treatment resulted in higher penetration force, lighter fillets (L*), and a clearly visible difference in color (ΔE values). However, in terms of flavor attributes, no clear differences between single and double freezing were found. In case of saithe (*Pollachius virens*), basically processed in the same way, it was found that the formaldehyde content was lower in single than double frozen fillets. Lightness and yellowness were higher in double-frozen fillets and refreezing also led to increased hardness and changes in other textural parameters. Moreover, double frozen fillets were not as fresh and their flavor was more stale and fishy. However, these changes were only valid for pre- and in-rigor processing. Since most fish are processed post rigor under commercial conditions, it was concluded that for battered and breaded products processed from refrozen fillet blocks, they were of similar quality as single-frozen fish [67]. As already mentioned above, Bøknæs et al. [58] concluded that the quality of double frozen cod in terms of loss of water upon cooking, WHC, and sensory quality of cooked samples were all of distinctly poorer quality compared with single-frozen cod.

### 2.7. Chilled Storage and Shelf-Life

The quality of fresh fish starts to deteriorate shortly after death, first by autolysis and then by bacterial spoilage. In the case of whitefish, loss of freshness by autolysis is the dominant factor for approximately the first week post mortem, provided adequate chilled storage conditions apply (core temperature approaches 0 °C). A gradual increase of bacterial numbers and spoilage subsequently occurs. In most cases, shelf life is determined by the number and type of bacteria present in the fish product, which is related to food safety. As a former recommendation, TVC should not exceed 5.0 × 10^5^ CFU g^−1^ in raw fish fillets, chilled or frozen [35]. However, todays EU-regulations (Commission Regulation no 2073/2005) do not give specific regulations for other parameters than histamine for whitefish. Companies are responsible to perform appropriate testing to validate that the production is safe, based on HACCP (Hazard Analysis and Critical Control Points) principles, and good hygiene practice.

As with frozen storage, it is important to maintain a stable, low temperature during chilled storage. Even a temporary increase in temperature for some hours can cause a significant reduction in shelf life [68]. To achieve the best possible market quality, it is rather obvious that fish should be frozen as soon as possible at sea, and after landing, the period between thawing and re-freezing at the processing plant should be as short as possible to minimize autolysis. A comprehensive guide to fresh fish quality is published by FAO. Among other issues, it covers topics like postmortem quality changes, shelf life of chilled fish, improved fish handling methods, and assessment of quality [69].

Others have reviewed the effect of modified atmosphere packaging (MAP) and vacuum packaging on shelf life and quality of fish [70,71,72,73]. It is well established that packaging both in vacuum or MAP can extend shelf-life of fish and fish products compared with storage in air [70,74,75,76]. However, packaging in MA does not seem to increase shelf life to any great extent compared with vacuum packaging [70]. The shelf life of MAP or vacuum-packed fish is dependent on the initial microbial status prior to handling as previously described, packaging materials, packaging method, gas mixture, gas to product volume ratio and storage temperature [55,70,74,77,78]. Carbon dioxide is the most important gas used in MAP of fish because it inhibits growth of many spoilage bacteria. Inhibition becomes more effective with increasing CO_2_-concentration in the atmosphere [70].

The main specific spoilage bacteria (SSBs) reported in cold water marine fish are *Pseudomonas* spp., *Shewanella putrefaciens* and *Photobacterium phosphoreum* [79]. Modified atmosphere has been shown to inhibit the normal spoilage flora [72], where the main bacteria present in vacuum and MA-packed fish are H_2_S-producing bacteria, including *S. putrefaciens* and *P. phosphoreum*, *Pseudomonas* sp., lactic acid bacteria, and *Enterobacteriacea* [80,81]. However, *P. phosphoreum* are reported the dominant spoilage organisms under MAP conditions, due to its ability to tolerate CO_2_ [80,82,83]. Studies have also reported that the growth of H_2_S-producing *Shewanella* was markedly reduced by CO_2_, and therefore, they were not responsible for spoilage and TMA formation in chilled MAP cod [84,85,86].

MA packaging can adversely affect the product quality, and especially high levels of CO_2_ can be associated with negative sensory characteristics. Besides quality and shelf life, solubility of CO_2_ also influences the risk of packaging collapsing [83,87]. Color, texture, and drip loss have all been reported to be negatively affected by high levels of CO_2_ [72,78,88]. The color of the belly flaps, cornea, gills, and the skin may be altered for fish stored in high CO_2_ concentrations [70]. Texture changes, including tissue softening are observed in fish fillets stored under pure CO_2_, although causes of texture changes associated with storage under MAP are not fully understood [72]. As discussed by Dewitt and Oliviera [72], texture changes probably are caused by high amounts of dissolved CO_2_, pH drop, excessive exudate, enzyme activity, or a combination of these. The authors found a direct correlation between the CO_2_ concentration and drip loss, since CO_2_ reduces WHC, although the amount of drip loss also depends on storage temperature and type of product.

Dalgaard et al. [80] studied the shelf life of cod fillets packed in MA (48% CO_2_) and in vacuum at 0 °C. The concentration of H_2_S-producing bacteria in MA package was very low. Compared with vacuum-packed fillets, a shelf-life extension of six to seven days was obtained with MAP. In 100% CO_2_, shelf-life was extended by two to three days. Shelf life was limited by poor texture and high drip-loss rather than by microbial activity. By comparison, the shelf-life of fresh cod fillets at 0–1 °C is 10–13 days [89]. Nguyen et al. [34] reported that the shelf life of cod bled after trawl capture, depended on whether the fish were bled in seawater (shelf life 10–11 days) or slurry ice (shelf life 13–14). The shelf lives were valid for fillets packed in EPS (expanded polystyrene) boxes with inner plastic bag stored at −1 ± 0.5 °C. In the case of ice storage of fresh cod from zero to ten days, drip loss was lower at 0 °C than at 4 or 7 °C. After ten days, the weight loss varied between 6 to 8% [90].

Regarding processing and packaging, recent innovations include combined application of MAP with other preservative factors, such as minimal processing or the addition of antioxidants and/or antimicrobial compounds. Smart packaging, including active packaging [91] and edible films and coatings are also innovative packaging technologies that have gained increased attention in recent years [92]. CO_2_-emitters, which develop CO_2_ gas after sealing the package, have shown promising results. Packaging of cod with an CO_2_-emitter in combination with MA, have shown a nearly doubling of shelf life compared with storage in vacuum [93].

## 3. Studies Comprising the Freeze-Chilling Chain

### 3.1. Freeze-Chilling Chain

For an overview of selected key processing and quality parameters assessed in connection with research covering the entire freezing-chilling chain (Figure 1), refer to Table 1. It is important to realize that the quality of fish is a very complex concept [94]. Hence, quality is best described by a number of parameters covering different chemical and physical aspects of the fish. To assess effects of treatment on product quality as shown in Table 1, it follows that the various quality parameters that the authors used to define quality should be taken into consideration when assessing their research.

#### 3.1.1. Fish Frozen at Sea

Rotabakk et al. [16] compared cod quality after trawl and longline catches. The fish were caught at the same time and location. After gutting and beheading, the fish were frozen pre-rigor in 25 kg blocks before frozen storage at −23 °C for about three months. The fish were thawed by a two-step process, seven hours in water with an initial temperature of 10 °C in a bin, followed by separation of single fish from the blocks and subsequent storage in ice water for nine hours. The fish were then filleted at about 0 °C and loins were cut, wrapped in Al-foil and stored for seven days at about 1 °C. Catching method had no effect on microbial activity and drip loss, whereas cod caught by trawl had lower pH and WHC, as well as higher levels of TMA. Protein denaturation was more pronounced in trawl fish and Catch Damage Index data showed that trawl fish had more damages than longline fish, although longlining resulted in gaffing damages. Color and overall sensory quality revealed poor bleed-out in case of trawled cod. Other assets of longline cod were better overall sensory quality, higher fillet lightness and firmness. After ice storage for seven days, the mean drip loss, 4.4 ± 1.8%, was similar for fish caught by trawl and longline. Otherwise, post-thawing ice storage resulted in decreases for the following quality attributes: sensory analyses, firmness, WHC, and lightness, whereas microbial growth, pH, and protein denaturation increased.

Quality of cod frozen pre rigor at sea (−40 °C), stored at −25 °C for about six weeks, thawed, and subsequently subjected to storage at 0–2 °C for 14 days was studied by Roiha et al. [95]. Special focus was put on the thawing process where fish blocks were thawed by three methods, in water (initial temperature 18 °C, fish-to-water ratio 1:4), with or without air circulation, and by contact thawing by a converted plate freezer. Circulation of air during thawing resulted in a more homogenous environment and more rapid thawing compared with no air circulation. With, or without air circulation, the flesh temperature reached 0 °C after about 210 and 380 min, respectively. In the converted plate freezer, 0 °C was reached after about 230 min. Despite differences in thawing time, there were no significant differences between thawing regimes, fillet quality was generally considered good, and there were no indications of impaired food safety. However, after six days on ice, the TVCs were as follows, 6.4 log CFU g^−1^ (with air circulation), 5.6 log CFU g^−1^ (without air circulation), and 6.9 log CFU g^−1^ (converted plate freezer). When all parameters were considered collectively, a post-freezing shelf life during of 10 to 14 days was obtainable (chilled storage). In another study, Roiha et al. [79] evaluated quality and safety of cod frozen pre-rigor on board a trawler. The fish were stored at −28 °C for nine weeks before thawing and subsequent storage in ice for six days. Once again, special attention was given to the thawing process where thawing was carried out in freshwater and re-used after passing a heat-exchanger. Two methods were assessed: (i) air circulation and continuous water flow at constant temperature, 10 °C for 4 h, and (ii) water at 10 °C for 2 h before temperature was lowered to −0.5 °C during a period of about 26 h. Subsequently, the fish were filleted and put in EPS boxes with plastic film between ice and fish until analysis six days post thawing. No significant differences were observed between the two temperatures during thawing and they both preserved good quality fish as determined by TVB-N, thawing loss, drip loss, cooking yield, sensory evaluation, textural properties, and microbiological analyses. It was concluded that as long as fish were frozen immediately after capture and subjected to adequate conditions during frozen storage, thawing, processing, and subsequent ice storage, it will be possible for the industry to supply the market with good quality and safe fish throughout the year.

Martinsdóttir and Magnússon [15] carried out an extensive study to reveal whether sea-frozen, thawed cod fillets were suitable for the “chilled” seafood market. The effects of pre- vs. postrigor freezing, frozen storage time up to 17 mo at −25 °C, and thawing in air at 0–1, 5, 8, and 22 °C) were studied. Thawing times varied between 17.5 to 4 h. Drip losses ranged from 0.6 to 1.8%, although no significant differences between thawing temperatures were found. However, to avoid post-thawing bacterial growth, thawing at the lowest temperature was recommended. Moreover, after two months, drip loss was about 1% and not different between pre- and postrigor fillets. During ice storage, pre-rigor fillets were judged by the sensory panel as fresher than postrigor fillets after two months of frozen storage. After 17 mo, however, there was no longer significant difference in freshness. Ice storage at 4 °C resulted in drier and tougher fillets than those stored at 0–1 °C. TVC and H_2_S bacterial counts were higher in postrigor fillets than in their prerigor counterparts. This was explained by 40 h storage in slurry as the fillets were passing through rigor mortis before freezing on board. Furthermore, pre rigor fillets were more tender than fillets frozen post rigor. Sensory assessment showed that keeping quality of iced cod fillets after two and six months of frozen storage was 12–14 days, 10–11 days after 12 mo, and 7 days after 17 mo. This study showed a significant advantage of freezing the fish before the onset of rigor mortis to maximize the storage quality after thawing.

Based on a rather comprehensive experimental design, Bøknæs et al. [13] studied a variety of factors that might affect the quality of Baltic Sea and Barents Sea cod. Their main goals were to study the use of MAP during frozen storage, different frozen storage periods and temperature regimes, cod from different fishing grounds, and the effects of addition of TMAO and NaCl on fillet quality. Fish from the Baltic Sea were transported chilled from sea to a local fish company where the fish were filleted and packed in MA about one day post capture before they were blast-frozen for 4 h until the core temperature reached −30 °C. Fish from the Barents Sea were headed, gutted, and bled at 4 °C for 30 min immediately after capture (trawler). Then, the fish were filleted pre rigor before they were packed in interleaved blocks, frozen in a horizontal plate freezer for 2 h at −30 °C and stored for ten weeks before transport to laboratory. Before continued frozen storage at the laboratory, frozen portions were packed in MAP (40% CO_2_ + 40% N_2_ + 20% O_2_) in Riloten bags and trays including absorbent drip pads where the gas:fish ratio was always greater than 1:2). Similar MAP was used for Baltic Sea cod. Frozen storage at the laboratory was carried out as follows −30 °C for eight weeks (Baltic Sea cod) including comparison with −20 °C (constant vs. fluctuating temperature), and for the Barents Sea cod −30 °C for 15 weeks. All MAP were thawed for 20 h at 5 °C. Subsequently, samples were chilled for 0, 8, 14, and 21 days at 2 °C (and, to some extent, for 14 days at 5 °C). Of all measured quality attributes (Table 1), only a significant increase of drip loss was observed as a result of application of MAP during frozen storage suggesting packages of cod fillets without MAP during frozen storage are more appropriate for trading thawed chilled cod fillets in MAP. Contents of TMAO and NaCl were considerably higher in cod from the Barents Sea than in cod from the Baltic Sea. It was shown that these substances protected against inactivation of *Photobacterium phosphoreum* during frozen storage, explaining the observed difference in spoilage pattern in fish from the two fishing grounds. Despite modest production of TMA in Baltic Sea fillets, it was concluded that this raw material for production of thawed MAP products was less suitable due to high drip loss during chilled storage.

In another study by Bøknæs et al. [101], the overall goal was to select key parameters in good manufacturing practice for production thawed chilled MAP fillets. The effects of frozen storage temperature (−20 vs. −30 °C), frozen storage period (3, 6, 9, and 12 mo) and chill storage periods up to 21 days at 2 °C were evaluated. Trawl-caught cod from a single haul were deheaded, gutted, bled, and filleted before freezing pre-rigor in blocks on board in an horizontal plate freezer until core temperature reached −25 °C after about 2 h. Subsequently, the fillet blocks were kept at −30 °C for ten weeks. At arrival to the laboratory, cod pieces (<100 g) were sawed in the frozen state and placed in trays, including absorbent drip pads packed in Riloten bags in a MA (40% CO_2_ + 40% N_2_ + 20% O_2_). Frozen storage then continued at −20 and −30 °C for 3, 6, 9, and 12 mo before thawing for 20 h at 5 °C before chilled storage for up to 21 days at 2 °C. Based on the quality parameters shown in Table 1, the authors concluded that frozen storage up to 12 mo had no significant effect on quality attributes. Furthermore, the shelf life of MAP cod fillets was not affected by time of frozen storage and was at least 14 days. Generally, the drip loss increased from about 5% after thawing up to about 12% after 21 days of chilled storage. It should be mentioned though that *P. phosphoreum* was inactivated during frozen storage at −20 °C and no growth of this microorganism was observed during the following chilled storage for 21 days. In case of fish stored at −30 °C, however, growth was observed after seven days of chilled storage, showing incomplete inactivation during the period of frozen storage.

Bacterial flora composition, TMA content, and sensory panel assessment of cod captured by trawl (TR) and longlining (LL) were reported by Magnússon and Martinsdóttir [54]. On board the trawler, the fish were bled, gutted, and frozen in a plate freezer after four days on ice, whereas on the longliner, the fish were bled and gutted, and later filleted, skinned, and frozen ashore in a tunnel freezer within 24 h after capture. Fish from both types of vessels were stored at −25 °C for 8 weeks (TR) or 1 day, 5, 14, 27, and 52 weeks (LL). In both cases, thawing took place at 15 °C until the core temperature reached 0 °C. Subsequently, ice-storage was carried out at 0–1 °C for up to three weeks of unfrozen and thawed whole cod (TR) and fillets (LL). The results showed that frozen storage for ≤5 weeks had little effect on bacterial counts, but after ≥14 weeks, total counts and TMAO-reducing bacteria were reduced in fillets. Sensory evaluation showed that frozen fillets never had as high scores as unfrozen fillets in the early phase of ice storage. However, after 10–12 days, when fillets were regarded as unacceptable, there was no longer any difference between unfrozen and frozen fillets.

#### 3.1.2. Chilled Transport of Fish before Freezing Ashore

Another approach to freeze-chilling is to bring the fresh catch ashore before freezing (Figure 1). This would typically imply ice storage of bled and gutted fish from anything between a few hours up to some days post mortem before the fish are processed and frozen at the plant. Unless the duration of transport from sea to plant is very short, this production strategy obviously implies that the freshness of fish at the point of freezing will be inferior to what can be achieved at sea and the benefits of freezing before rigor onset will be lost.

Chilled cod, captured at sea, were transported to a fish processor where Bøknæs et al. [55] exposed cod fillets to air (0 °C) for one and eight days before packing in MA (60% CO_2_ + 40% N_2_) and blast freezing until core temperature reached −30 °C. The fillets were then subjected to frozen storage for six weeks at −20 or −30 °C before they were thawed in air for 14 h at 5 °C. Subsequently, the packages were stored at 2 °C for up to 17 days. *P. phosphoreum*, a specific spoilage organism in fresh MAP cod, was found in levels of 2.3 and 5.8 CFU g^−1^ after one and eight days of chilled storage in air. After frozen storage, the levels were at both storage temperatures reduced below the limit of detection. Furthermore, WHC was significantly reduced due to freezing. Significant growth of *P. phosphoreum* and production of TMA during chilled storage at 2 °C were only observed in cod fillets stored for eight days prior to packing in MA before freezing at −30 °C. After thawing of MAP cod fillets, frozen storage odor and taste were clearly most pronounced in fillets stored for eight days before freezing. It was concluded that the combination of using fish immediately frozen at sea, without ice storage before freezing, with MAP technology can improve product quality where MAP delays microbiological spoilage of the thawed products.

Fresh cod fillets were transported to laboratory one day after capture and processing. Here, the fillets were packed in MA (40% CO_2_ + 60% N_2_) vs. (40% CO_2_ + 40% N_2_ + 20% O_2_) before blast-freezing at −40 °C until core temperature reached −20 °C [102]. The packages were stored at −20 °C for two months before the spoilage characteristics and shelf life were determined after thawing in air for 16 h at 5 °C and storage at 2 °C for 20 days. A comparison was made with fresh fillets in MAP. The shelf life of fresh fillets was 11–12 days. Regarding their frozen/thawed counterparts, the shelf life was extended by more than 20 days, most likely as a result of inactivation of the spoilage bacterium *P. phosphoreum* during frozen storage. Furthermore, no significant production of TMA and little amine odor and taste were detected in thawed MAP fillets after chilled storage for 20 days. It was concluded that frozen MAP provides a more stable product and allows much greater flexibility for production and distribution. On the other hand, thawed fillets had a slight increase in DMA, a weak frozen storage flavor, as well as a larger drip loss.

Freeze-chilling of vacuum-packed cod loins was studied by Jensen et al. [98]. High quality cod (Grade E) were bought and filleted immediately after landing before transport in ice to laboratory where loins were packed in individually in vacuum before they were blast frozen at −40 °C, subsequently stored at −30 °C for 46–66 days, thawed slowly at 2 °C, and chilled from 0 up to 12 days at 2 °C. The products exhibited a consistent high sensory quality for six days of chilled storage before the sensory quality decreased gradually. If quality is not to be compromised, the maximum storage time was considered ten days. Drip loss increased from about 4 to 8% during ice storage for 0 to 12 days. It was concluded that supplying the market with freeze-chilled meal elements, high-quality cod products are practically applicable.

Vyncke [103] filleted and skinned cod at a wholesaler’s premises six days post capture before packing in vacuum, freezing in an air blast freezer at −40 °C prior to frozen storage at −28 °C for 1 week, 3, 6, and 12 mo. After thawing for 2 h in a circulating water bath at 18 °C, vacuum packages were removed and the fillets were stored in ice for up to 13 days. Results were compared with fresh fillets from the same batch iced immediately upon arrival from sea. Based on organoleptic tests, unfrozen fillets reached their limit of acceptability after about eight days, whereas the frozen/thawed fillets prolonged shelf life with two days (1 week frozen storage) and three to four days (3 to 12 mo frozen storage). Furthermore, frozen storage resulted in a slight increase in firmness after six months. Cold-store flavor and odor were absent in all fish groups and no discoloration was observed. The results were explained by reduced bacterial counts in frozen/thawed samples and slower increases in pH, TVB, TMA, and Total Volatile Acids Number (VAN). Notably, one week of frozen storage did not alter the spoilage pattern relative to fresh fillets.

After about three days post capture (ice storage), Fagan et al. [99] combined freeze-chilling of whiting (*Merlangius merlangus*) portions/fillets with MAP (30% N_2_, 40% CO_2_, 30% O_2_). The treatment was compared with fillets packed in trays with plastic film. Both types of packages were blast-frozen at −35 °C for 2.5 h, followed by storage at −30 °C for three days, before thawing overnight at 2–4 °C. Subsequently, the packages were subjected to chilled storage (2–4 °C) for up to five days. The quality of the MAP product was regarded acceptable after five days, whereas storage in air resulted in a shelf life of three days. Color changed during storage as portions gradually became more yellow, although less so in MAP than in air. MAP had no effect on moisture content and samples packed in air had less gravity drip than samples in MAP. Storage time had no effect on gravity drip. In contrast, centrifugal drip increased during storage. Furthermore, MAP had no effect on TVBN or TMA, although the compounds increased progressively during chilled storage. Microbial counts also increased, where MAP counts were lower than in air. It was concluded that freeze-chilled MAP products performed well and they have logistic benefits in product distribution and retailing.

In another test with whiting, Fagan et al. [100] compared the following treatments: (a) fresh (about 3 days post mortem); (b) chilled (3 days at 4 °C); (c) air-blast frozen at −35 °C for 3 h before storage at −30 °C for three days followed by thawing overnight at 4 °C; (d) freeze-chilled: frozen, stored and thawed as (c) followed by chilled storage at 4 °C for three days. Frozen fish exhibited lower scores (better odor) than fresh and freeze-chilled fish and there was no difference between the latter two treatments. Acceptability by a sensory panel was highest for fresh fish followed by frozen, chilled, and freeze-chilled fish, although there was no statistical difference between chilled and freeze-chilled fish. Whiteness and redness were unaffected by treatment, but freeze-chilled fish were more yellow. Regarding texture (springiness), only fresh fish were different by being less springy. Gravity drip was significantly different as 1.0 (chilled), 9.0 (frozen), and 6.0% (freeze-chilled). Chilled and freeze-chilled fish exhibited higher TVBN, TMA, and TVC values than fresh and frozen fish, whereas water content was not affected significantly by treatment. It was concluded that freeze-chilling is a suitable technology for prepacked whiting.

#### 3.1.3. Other Freeze-Chilling Studies

Another source of providing fish for freeze-chilling can be from the live fish market exampled by Li et al. [96] who obtained common carp (*Cyprius carpio*) before the fish were stunned, gutted, washed, and packed in polyethylene bags. Sensory scores, quality attributes and microbial communities in white and dark muscles were analyzed during chilled storage (4 °C for 10 days) alone (control group) compared with freeze-chilling where the fish had been frozen and stored at −20 °C for 4 weeks before chilled storage under similar conditions as the control group. Based on sensory assessment, the shelf life of chilled white and dark muscle of control fish was eight and ten days, respectively. Both types of freeze-chilled muscles had a shelf-life of eight days. By then, TVC exceeded the limit of 7.00 log CFU g^−1^. Significant differences in the microbial profiles between chilled and freeze-chilled samples were revealed.

Yin et al. [97] studied the effect of fresh storage, freezing alone, and freeze-chilling on the quality of grass carp (*Ctenopharyngodon idellus*) fillets. Fish, bought live at the market, were killed, filleted, and packed in PVC bags before they were subjected to the following treatments: (1) chilled storage at 4 °C for six days, (2) frozen storage at −40 °C for 12 h, then at −20 °C for five days, and (3) freeze-chilled storage at −40 °C for 12 h, then at −20 °C for five days, followed by chilled storage at 4 °C for four days. Frozen fish were thawed in refrigerator at 2 °C for 12 h. In addition, fresh fish were tested at Day 0. Among the findings were significant differences in case of drip loss in the following order: (3) > (2) > (1), probably due to reduced WHC caused by protein denaturation. Furthermore, total sensory scores showed higher quality for (1) than (3) within four days of chilled storage. Fillets generally became lighter, less red and more yellow with increasing storage time relative to Day 0 fillets. Overall, it was concluded that chilled fillets showed higher sensory score and better color and texture characteristics than frozen and freeze-chilled fillets within three days. However, if transport to market extends five days, freeze-chilling was considered a good alternative where the product can be held frozen until required by market demands. Chilled fillets (1) should be retailed and processed within four days.

## 4. Conclusions

Due to the large variation in experimental designs, choice of parameters defining product quality, experimental conditions, and parameters, direct comparisons of the research shown in the present review is hardly possible. Instead, the research should be viewed as a collection of case studies.

It is well established that the intrinsic quality of whitefish depends on factors such as season, fishing ground, and fish size. The type of fishing gear used can have a significant effect on whitefish quality, predominantly on the external appearance of fish. While recent research has shown that modified fishing gear can indeed improve quality, the methods cannot yet be applied on a commercial scale since they must be approved by the relevant authorities in the respective countries where size selectivity would also be an issue to consider.

Current fishing methods for capture of whitefish inherently result in some degree of handling stress where several biochemical pathways are activated triggering autolysis at an early stage. However, the effect on whitefish quality seems to be modest compared with other, more active fish species. For the time being, it seems rather far-fetched to imagine that whitefish can be caught with a minimum of exposure to stress.

The introduction of electrical immobilization of fish on vessels makes it possible to facilitate proper bleed-out and thereby improve fillet quality in terms of color characteristics. For large catch sizes, the method can be combined with keeping the fish in water-filled tanks on board to make sure the entire catch will stay alive until they are stunned and bled. It can be expected that the same system also makes it possible to process and freeze the entire catch pre rigor, an important concept to obtain high-quality fish products.

In line with a previous review [2], there is no clear consensus whether slow versus rapid freezing rates have unambiguous effects on fish quality. However, a recent study confirms that when fish are frozen pre-rigor, no differences in microstructure and quality were observed when a comparatively slow freezing rate was compared with rapid freezing rates. This verifies that freezing rate is of less importance to obtain a high-quality product when fish are frozen before rigor onset. Glazing of the fish before frozen storage is clearly a method to preserve quality, although this should be weighed against the gain in weight of the product.

Regarding frozen storage, it is not straight-forward to give specific recommendations regarding *storage time* × *temperature* conditions to optimize whitefish quality due to the considerable differences in experimental conditions in previous research. Generally, it is recommended the storage temperature of lean fish should be between −24 and −30 °C. There are also indications that storage temperatures below −40 °C may not have an overall substantial positive effect on quality. Frozen storage time should be taken into consideration since long-term storage can reduce bacterial counts or alter the bacterial composition, which in turn will affect shelf life when fish are subjected to chilled storage after thawing. On the other hand, prolonged frozen storage leads to deterioration of quality such as muscle toughening.

Appropriate thawing conditions depend on preceding processing history. In cases when the fish are frozen pre-rigor, thaw rigor might occur, which have detrimental effects on quality. Thaw rigor can be avoided by either subjecting the fish to long-term frozen storage before thawing or by slow thawing. Otherwise, there is no consistency in the literature as to whether whitefish should preferably be thawed slowly or rapidly. Despite the considerable differences in thawing temperature–time regimes, as well as in type of equipment used, research results are often regarded satisfactory in terms of post-thawing product quality. Such findings may be interpreted as whitefish are quite robust when it comes to thawing conditions and effect on quality. Since many research papers study thawing methods after the fish have gone through preceding steps in the value chain, it could, on the other hand, well be that substantial changes in quality (issues described above) have already occurred, before the thawing studies were conducted. Obviously, such studies are nevertheless valid in their own right for assessing the effect of the entire value chain on quality. However, if the goal is to study a certain thawing method *per se*, it is questionable to what extent such thawing studies are valid. To evaluate the effect of thawing alone, it is recommended to use pre-rigor fish (to minimize the effect of freezing on quality at a cellular level) and to basically offset the effect of frozen storage by initiating the thawing process as soon as the desired core temperature is reached. In this way, it should be possible to obtain better estimates of quality (before and after thawing as a unit operation) to assess how well a thawing method is able to preserve quality.

When double-frozen whitefish are compared with single-frozen ones, consensus is that refreezing results in less favorable product quality. Fillet properties affected adversely are typically drip loss, WHC, color, and sensory quality. However, the quality of double frozen fish can still be regarded as acceptable and is perhaps more suitable as a raw material for processed fish products.

Concerning chilled storage and shelf life, it is well established that good chilling practices are important. In practice, this usually means keeping core temperatures approaching 0 °C. The autolytic phase (loss of freshness) then approximately occurs during the first week post mortem. Subsequently, microbial degradation processes gradually become more dominant and defines quality and food safety. Under such regimes, the shelf life of cod is typically within the range of 10 to 14 days post mortem. It is well established that packaging both in vacuum or MAP can extend shelf life of fish and fish products compared with storage in air. However, the shelf life extension depends on initial quality, packaging method and type of material, as well as storage conditions. When the pre-freezing storage period of captured fish, the unfrozen vs. frozen status of the fish, and packing technology are considered, the estimated shelf lives of freeze-chilled cod seem to be largely comparable to traditional chilled storage of fresh or frozen/thawed whitefish.

The freeze-chilling concept is merely a combination of the consecutive processing steps described above. Hence, best practice for optimizing each processing step in terms of product quality is also relevant for optimizing the freeze-chilling chain. Some effects on quality of previous processing steps may only become apparent during the post-thawing chilled storage phase. Examples are alteration of bacteria flora and inactivation of certain spoilage bacteria as a result of prolonged frozen storage or MA-packaging. Consequently, the shelf life of freeze-chilled fish can therefore be prolonged compared with traditional ice storage of unfrozen fish, or in cases where the duration of frozen storage is comparatively short. On the other hand, prolonged frozen storage seems to off-set the beneficial effects of freezing the fish pre-rigor (relevant for single-frozen fish only).

When it comes to an evaluation of the freeze-chilling concept for whitefish, previous research has shown that the method can produce fish products of good quality and adequate food safety provided good processing conditions are adhered to. Clearly, the method also offers logistical advantages. When compared quality-wise with fresh fish (not frozen), the quality of freeze-chilled fish cannot be expected to be quite as good as unfrozen fish during the first few days of chilled storage post mortem. Thus, when transport time of fresh fish to market exceeds this period, the freeze-chilling concept can be an advantageous marketing strategy to consider when product quality alone is considered. Furthermore, fish in MAP and vacuum are considered as stable products, convenient and flexible for market distribution of high-quality products.

## Figures and Tables

**Table 1:** Freeze-chilling of fish: Overview of fish species, initial freshness (day 0 is defined as day of capture and death), freezing temperatures, frozen and chilled storage temperatures and durations for each step in the value chain, type of packaging, as well as parameters used for assessment of quality for each study.

Species	Freshness at Freezing	Freezing Conditions	Frozen Storage Conditions	Thawing Conditions	Packaging	Chilled Storage	Quality Analyses	Shelf Life	Reference
Atlantic cod	Day 0 Pre-rigor *	−40 °C	−25 °C for ≈ 6 weeks	Water (initial temp. 18 °C, fish-to-water ratio 1:4) with and without air circulation or by contact thawing in a converted plate freezer	After thawing, storage over-night at 0 °C, before filleting and packaging in plastic film	14 days at 0–2 °C	Bacteria (*E.Coli*, *Listeria*, H_2_S-bacteria, coliforms, thermo-tolerant coliforms), CO, CY, DL, PC, SE, TX, TVBN, TVC, WHC	10–14 days	Roiha et al. [95]
Atlantic cod	Day 0 Pre-rigor *	−40 °C	−28 °C for 9 weeks	Two methods: (i) air circulation and continuous water flow at 10 °C (constant temp.) for 4 h, (ii) water at 10 °C for 2 h before water temp. was lowered to −0.5 °C for ≈ 26 h.	After thawing, filleting and packaging (EPS boxes) with plastic film between ice and fish.	6 days at 2.9 ± 0.6 °C	Bacteria (*E. coli*, *Listeria*, H_2_S-bacteria, coliforms, thermo-tolerant coliforms), blood spots, CY, DL, pH, QIM (fillets), redness, TVBN, TVC, TX, WC	NA (>6 days)	Roiha et al. [79]
Carp	Day 0 *	−20 °C (vs fresh control at 4 °C)	−20 °C for 4 weeks	NA	Fillets in polyethylene bags	8 days at 4 °C	Bacterial DNA & rRNA, DL, SP, TVBN, TVC, VOC	8 days (white muscle) 10 days (red muscle)	Li et al. [96]
Carp	Day 0 *	−40 °C for 12 h	Control: Day 0 (1) 4 °C for 6 days; (2) −20 °C for 5 days (3) −20 °C for 5 days before chilled storage for 4 days.	2 °C for 12 h	Fillets in PVC bags	4 to 6 days at 4 °C	CO, DL, protein content, SE, TAC, TVBN, TX	NA	Yin et al. [97]
Atlantic cod	Pre-rigor*	ND (plate freezer)	−23 °C for about 3 mo	Fish blocks thawed in a two-step process: (a) 7 h in water (initial temperature 10 °C) in bin, followed by (b) separation of single fish from blocks and storage in ice water for 9 h	After thawing, loins wrapped in Al-foil	Up to 7 days at 0–2 °C	APC, bacteria (psychrotrophic, *P. phosphoreum*), CO, DL, DSC, FI, pH, SE, TMA, TX (firmness), WHC	NA	Rotabakk et al. [16]
Atlantic cod	Not stated, processed on day of arrival (unfrozen) to port	−40 °C	−30 °C for 46 to 66 days	Slowly at 2 °C	Individual loins in vacuum	0 to 12 days	DL, SE, DM, LHC	NA (>6 days)	Jensen et al. [98]
Whiting	3 days fillets	−35 °C for 2.5 h	−30 °C for 3 days	Overnight at 2–4 °C	Individual fillet/portions in trays: air vs. MAP	5 days at 2–4 °C	CO, DL, SP, TMA, TVBN, TVC, TX, WC	NA (>5 days)	Fagan et al. [99]
Whiting	3 days fillets	−35 °C for 2.5 h	−30 °C for 3 days	Overnight at 4 °C	Individual fillet/portions in trays: aerobic conditions	3 days at 4 °C	CO, DL, SP, TMA, TVBN, TVC, TX, WC	NA (>3 days)	Fagan et al. [100]
Atlantic cod	Pre-rigor *	Until core temperature reached −25 °C after 2 h	(i)−30 °C for 10 weeks (on vessel) followed by (ii) −20 °C vs. −30 °C (still as blocks) for 3, 6, 9 and 12 mo	MAP thawed for 20 h at 5 °C before chilled storage	Fillets packed with interleave plastic film frozen as blocks. Cod pieces (<100 g) sawed in frozen state, placed in trays with absorbent, MA-packed in Riloten bags (40% CO_2_, 40% N_2_, 20% O_2_)	0–21 days at 2 °C	Bacteria (*P. phosphoreum)*, DL, DMA, FA, NaCl, pH, TMA, TMAO, sensory panel (odor, taste, juiciness), TVC, WHC	14–21 days at −30 °C >21 days at −20 °C	Bøknæs et al. [101]
Atlantic cod	Pre-rigor *	(i) < 2 h + 6 fillets at −196 °C, (ii) One batch in SW-slurry at 3–6 °C for 40 h post rigor, before frozen in plate freezer + 6 fillets at −196 °C	−25 °C for 2, 6, 12 and 17 mo	(i) Thawing trial after 2 mo: pre- vs. post-rigor fillets: in air at 0–1, 5, 8 and 22 °C, (ii) Overnight at 0–1 °C, then at room temp. for 2–3 h until core temperature was 0 °C.	Fillets	(i) Only after 17 mo storage: 0–15 days at 4 and 0 °C, (ii) Thawing trial: 9 days at 0–1 °C	Bacteria (H_2_S), DL, pH, SAP, TMA, TVC	12–14 days (2–6 mo) ** 10–11 days (12 mo) ** 7 days (17 mo) **	Martinsdottir and Magnusson [15]
Baltic Sea cod and Barents Sea cod	Baltic Sea cod: Chilled transport from sea to local fish company, filleted and packed in MA 1 days post capture Barents Sea cod: pre-rigor filleted on board	Baltic Sea cod: Blast-frozen for 4 h at −30 °C Barents Sea cod: processed on trawler, frozen in plate freezer for 2 h, stored at −30 °C for 10 weeks before transport to lab. Before refreezing at lab., portions were packed in MAP, stored at −30 °C for another 5 weeks	Baltic Sea cod: 30 °C for 8 weeks and −20 °C (constant vs. fluctuating temperature) Barents Sea cod: −30 °C for 15 weeks	MAP thawed for 20 h at 5 °C	MAP (40% CO_2_, 40% N_2_, 20% O_2_) in Riloten bags and trays including absorbent using gas:fish ratio > 1:2.	(i) 0–21 days at 2 °C (ii) 14 days at 5 °C	Amine odor, Bacteria (*P. phosphoreum*), DL, juiciness, NaCl, TMA, TMAO, TVC	NA	Bøknæs et al. [13]
Atlantic cod	After chilled transport from sea to fish company	Blast-frozen until core temp reached −30 °C	−20 °C or −30 °C for 6 weeks	In air for 14 h at 5 °C	MAP (60% CO_2_, 40% N_2_) in Riloten bags, fish:gas = 1:2, fillets also wrapped in PE and stored aerobically at 0 °C for 7 days until packed in MA	17 days at 2 °C	Bacteria (*P. phosphoreum*), SP, TMA, TMAO	NA	Bøknæs et al. [55]
Atlantic cod	1 day fillets	−40 °C for 3.5 h (MAP)	−20 °C for 2 mo	In air at 5 °C for 16 h	MAP (40% CO_2_, 60% N_2_) vs. (40% CO_2_, 40% N_2_, 20% O_2_)	20 days at 2 °C	DMA, DL, SB, SP, TMA, TVC	11–12 days (fresh MAP) >20 days (thawed MAP)	Guldager et al. [102]
Atlantic cod	Fish from (I) trawler: whole gutted fish (II) longliner: gutted, filleted, skinned ashore within 24 h post capture, subsequently (a) fresh fillets on ice, or (b) fillets frozen	(1) plate freezer (vessel) after 4 days on ice, (2) tunnel freezer (ashore)	−25 °C for (I) 8 weeks, (II) 1 day, 5, 14, 27 and 52 weeks	(I) and (II) at 15 °C	Whole fish and fillets	0–1 °C for up to 3 weeks as unfrozen and thawed whole cod (I), or 3 weeks as fillets (II)	Bacteria (flora composition, H_2_S), SP, TMA, TMAO, TVB, TVC	10−12 days	Magnusson and Martinsdottir [54]
Atlantic cod	6 days fillets	−40 °C	−28 °C for 1 week and 3, 6 and 12 mo	Circulating water at 18 °C	In vacuum before freezing and packaging removed before ice storage	13 days	pH, SP, TMA, TVBN, TVC, VAN	8 days (fresh) 10 days (1 week) ** 11–12 days (3–12 mo) **	Vyncke [103]

* rigor state not determined, but pre-rigor condition highly probable based on time post mortem when freezing commenced; ** frozen storage; APC (aerobic plate counts); CO (color); CY (cooking yield); DL (drip loss); DMA (dimethylamine); FI (fillet index); PC (proximate composition); SB (spoilage bacteria); SE (sensory evaluation); SP (sensory panel); TAC (total aerobic counts); TMA (trimethylamine); TVBN (total volatile base nitrogen); TVC (total viable count); TX (texture); VAN (total volatile organic compounds); VOC (volatile organic compounds); WC (water content); NA (not assessed).

## Data Availability

Not applicable.

## References

[1] Nicholson F.J. (1982). The Freezing Time of Fish.

[2] Hedges N., Bremner H.A. (2002). Maintaining the quality of frozen fish. Safety and Quality Issues in Fish Processing.

[3] Kolbe E., Kramer D. (2007). Planning for Seafood Freezing.

[4] Gökoğlu N., Yerlikaya P. (2015). Seafood Chilling, Refrigeration and Freezing: Science and Technology.

[5] Svendsen E.S., Widell K.N., Tveit G.M., Nordtvedt T.S., Uglem S., Standal I., Greiff K. (2021). Industrial methods of freezing, thawing and subsequent chilled storage of whitefish. J. Food Eng..

[6] Backi C.J. (2017). Methods for (industrial) thawing of fish blocks: A review. J. Food Process. Eng..

[7] Cai L., Cao M., Regenstein J., Cao A. (2019). Recent Advances in Food Thawing Technologies. Compr. Rev. Food Sci. Food Saf..

[8] Nagarajarao R.C. (2016). Recent Advances in Processing and Packaging of Fishery Products: A Review. Aquat. Procedia.

[9] Gonçalves A.A., Blaha F. (2011). Cold chain in seafood industry. Refrigeration: Theory, Technology and Applications.

[10] Maccallum W.A., Jaffray J.I., Churchill D.N., Idler D.R. (1968). Condition of Newfoundland Trap-Caught Cod and Its Influence on Quality After Single and Double Freezing. J. Fish. Res. Board Can..

[11] Castell C.H., Kreuzer R. (1971). Some fundamental problems in the quality assessment of fishery products. Fish Inspection and Quality Control.

[12] Love L.M. (1988). The Food Fishes, Their Intrinsic Variation and Practical Implications.

[13] Bøknæs N., Østerberg C., Sørensen R., Nielsen J., Dalgaard P. (2001). Effects of Technological Parameters and Fishing Ground on Quality Attributes of Thawed, Chilled Cod Fillets Stored in Modified Atmosphere Packaging. LWT.

[14] Botta J.R., Bonnell G., Squires B.E. (1987). Effect of Method of Catching and Time of Season on Sensory Quality of Fresh Raw Atlantic Cod (*Gadus morhua*). J. Food Sci..

[15] Martinsdottir E.M., Magnusson H. (2001). Keeping Quality of Sea-Frozen Thawed Cod Fillets on Ice. J. Food Sci..

[16] Rotabakk B.T., Skipnes D., Akse L., Birkeland S. (2011). Quality assessment of Atlantic cod (*Gadus morhua*) caught by longlining and trawling at the same time and location. Fish. Res..

[17] Margeirsson S., Jonsson G.R., Arason S., Thorkelsson G. (2007). Influencing factors on yield, gaping, bruises and nematodes in cod (*Gadus morhua*) fillets. J. Food Eng..

[18] Botta J.R., Bonell G. (1989). Causes of reduced quality of fresh Atlantic cod (*Gadus morhua*) caught by otter trawl. Proceedings of the World Symposium on Fishing Gear and Fishing Vessel Design.

[19] Veldhuizen L., Berentsen P., de Boer I., van de Vis J., Bokkers E. (2018). Fish welfare in capture fisheries: A review of injuries and mortality. Fish. Res..

[20] Tveit G.M., Sistiaga M., Herrmann B., Brinkhof J. (2019). External damage to trawl-caught northeast arctic cod (*Gadus morhua*): Effect of codend design. Fish. Res..

[21] Digre H., Hansen U.J., Erikson U. (2010). Effect of trawling with traditional and ‘T90′ trawl codends on fish size and on different quality parameters of cod *Gadus morhua* and haddock *Melanogrammus aeglefinus*. Fish. Sci..

[22] Brinkhof J., Olsen S.H., Ingólfsson Ó.A., Herrmann B., Larsen R.B. (2018). Sequential codend improves quality of trawl-caught cod. PLoS ONE.

[23] Sistiaga M., Herrmann B., Brinkhof J., Larsen R.B., Jacques N., Santos J., Gjøsund S.H. (2020). Quantification of gear inflicted damages on trawl-caught haddock in the Northeast Atlantic fishery. Mar. Pollut. Bull..

[24] Brinkhof J., Herrmann B., Sistiaga M., Larsen R.B., Jacques N., Gjøsund S.H. (2021). Effect of gear design on catch damage on cod (*Gadus morhua*) in the Barents Sea demersal trawl fishery. Food Control.

[25] Brinkhof J., Larsen R.B., Herrmann B., Olsen S.H. (2018). Assessing the impact of buffer towing on the quality of Northeast Atlantic cod (*Gadus morhua*) caught with a bottom trawl. Fish. Res..

[26] de Haan D., Fosseidengen J.E., Fjelldal P.G., Burggraaf D., Rijnsdorp A.D. (2016). Pulse trawl fishing: Characteristics of the electrical stimulation and the effect on behaviour and injuries of Atlantic cod (*Gadus morhua*). ICES J. Mar. Sci..

[27] Digre H., Erikson U., Misimi E., Standal I.B., Gallart-Jornet L., Riebroy S., Rustad T. (2011). Bleeding of Farmed Atlantic Cod: Residual Blood, Color, and Quality Attributes of Pre- and Postrigor Fillets as Affected by Perimortem Stress and Different Bleeding Methods. J. Aquat. Food Prod. Technol..

[28] Olsen S.H., Tobiassen T., Akse L., Evensen T.H., Midling K.Ø. (2013). Capture induced stress and live storage of Atlantic cod (*Gadus morhua*) caught by trawl: Consequences for the flesh quality. Fish. Res..

[29] Misimi E., Erikson U., Digre H., Skavhaug A., Mathiassen J. (2008). Computer Vision-Based Evaluation of Pre- and Postrigor Changes in Size and Shape of Atlantic Cod (*Gadus morhua*) and Atlantic Salmon (*Salmo salar*) Fillets during Rigor Mortis and Ice Storage: Effects of Perimortem Handling Stress. J. Food Sci..

[30] Erikson U., Digre H., Misimi E. (2011). Effects of Perimortem Stress on Farmed Atlantic Cod Product Quality: A Baseline Study. J. Food Sci..

[31] Erikson U., Tveit G., Bondø M., Digre H. (2019). On-board Live Storage of Atlantic Cod (*Gadus morhua*): Effects of Capture Stress, Recovery, Delayed Processing, and Frozen Storage on Fillet Color Characteristics. J. Aquat. Food Prod. Technol..

[32] Digre H., Rosten C., Erikson U., Mathiassen J.R., Aursand I.G. (2017). The on-board live storage of Atlantic cod (*Gadus morhua*) and haddock (*Melanogrammus aeglefinus*) caught by trawl: Fish behaviour, stress and fillet quality. Fish. Res..

[33] Olsen S.H., Joensen S., Tobiassen T., Heia K., Akse L., Nilsen H.A. (2014). Quality consequences of bleeding fish after capture. Fish. Res..

[34] Van Nguyen M., Karlsdottir M.G., Olafsdottir A., Bergsson A.B., Arason S. (2013). Sensory, microbiological and chemical assessment of cod (*Gadus morhua*) fillets during chilled storage as influenced by bleeding methods. Int. J. Nutr. Food Eng..

[35] Tobiassen T., Akse L., Midling K., Aas K., Dahl R., Eilertsen G., Luten J.B. (2006). Quality of farmed fish: The effect of pre rigor processing of cod (*Gadus morhua* L.) on quality and shelf life. Seafood Research from Fish to Dish: Quality, Safety and Processing of Wild and Farmed Seafood.

[36] Erikson U., Grimsmo L., Digre H. (2021). Establishing a Method for Electrical Immobilization of Whitefish on Board Fishing Vessels. J. Aquat. Food Prod. Technol..

[37] Heen E. (1982). Developments in chilling and freezing of fish. Int. J. Refrig..

[38] Anderssen K.E., Syed S., Stormo S.K. (2021). Quantification and mapping of tissue damage from freezing in cod by magnetic resonance imaging. Food Control.

[39] Erikson U., Kjørsvik E., Bardal T., Digre H., Schei M., Søreide T.S., Aursand I.G. (2016). Quality of Atlantic Cod Frozen in Cell Alive System, Air-Blast, and Cold Storage Freezers. J. Aquat. Food Prod. Technol..

[40] Offer G., Cousins T. (1992). The mechanism of drip production: Formation of two compartments of extracellular space in musclePost mortem. J. Sci. Food Agric..

[41] Connell J.J., Howgate P.F. (1968). Sensory and objective measurements of the quality of frozen stored cod of different initial freshnesses. J. Sci. Food Agric..

[42] Mørkøre T., Lilleholt R. (2007). Impact of freezing temperature on quality of farmed atlantic cod (*Gadus morhua* L.). J. Texture Stud..

[43] Haard N.F., Bligh E.G. (1990). Biochemical reactions in fish muscle during frozen storage. Seafood Science and Technology.

[44] Mackie I.M. (1993). The effects of freezing on flesh proteins. Food Rev. Int..

[45] Jessen F., Nielsen J., Larsen E. (2013). Chilling and Freezing of Fish. Seafood Processing.

[46] Biglia A., Comba L., Fabrizio E., Gay P., Aimonino D.R. (2016). Case Studies in Food Freezing at Very Low Temperature. Energy Procedia.

[47] Leblanc E.L., Leblanc R.J., Blum I.E. (1988). Prediction of Quality in Frozen Cod (*Gadus morhua*) Fillets. J. Food Sci..

[48] Kim Y.J., Heldman D.R. (1985). Quantitative analysis of texture change in cod muscle during frozen storage. J. Food Process. Eng..

[49] Careche M., Del Mazo M.L., Torrejón P., Tejada M. (1998). Importance of Frozen Storage Temperature in the Type of Aggregation of Myofibrillar Proteins in Cod (*Gadus morhua*) Fillets. J. Agric. Food Chem..

[50] Badii F., Howell N.K. (2002). Changes in the texture and structure of cod and haddock fillets during frozen storage. Food Hydrocoll..

[51] Burgaard M.G., Jørgensen B.M. (2010). Effect of Temperature on Quality-Related Changes in Cod (*Gadus morhua*) During Short- and Long-Term Frozen Storage. J. Aquat. Food Prod. Technol..

[52] Lee J., Park J.W. (2016). Pacific whiting frozen fillets as affected by postharvest processing and storage conditions. Food Chem..

[53] Baixas-Nogueras S., Bover-Cid S., Veciana-Nogués M.T., Vidal-Carou M.C. (2007). Effects of previous frozen storage on chemical, microbiological and sensory changes during chilled storage of Mediterranean hake (*Merluccius merluccius*) after thawing. Eur. Food Res. Technol..

[54] Magnússon H., Martlnsdóttlr E. (1995). Storage Quality of Fresh and Frozen-thawed Fish in Ice. J. Food Sci..

[55] Bøknæs N., Østerberg C., Nielsen J., Dalgaard P. (2000). Influence of Freshness and Frozen Storage Temperature on Quality of Thawed Cod Fillets Stored in Modified Atmosphere Packaging. LWT.

[56] Aubourg S.P., Medina I. (1999). Influence of storage time and temperature on lipid deterioration during cod (*Gadus morhua*) and haddock (*Melanogrammus aeglefinus*) frozen storage. J. Sci. Food Agric..

[57] Badii F., Howell N.K. (2002). A comparison of biochemical changes in cod (*Gadus morhua*) and haddock (*Melanogrammus aeglefinus*) fillets during frozen storage. J. Sci. Food Agric..

[58] Boknes N., Guldager H.S., Sterberg C., Nielsen J. (2001). Production of High Quality Frozen Cod (*Gadus morhua*) Fillets and Portions on a Freezer Trawler. J. Aquat. Food Prod. Technol..

[59] Cappeln G., Jessen F. (2001). Glycolysis and ATP Degradation in Cod (*Gadus morhua*) at Subzero Temperatures in Relation to Thaw Rigor. LWT.

[60] Cappeln G., Nielsen J., Jessen F. (1999). Synthesis and degradation of adenosine triphosphate in cod (*Gadus morhua*) at subzero temperatures. J. Sci. Food Agric..

[61] Stroud G.D. (1969). Rigor in Fish: The Effect on Quality.

[62] Hurling R., Mcarthur H. (1996). Thawing, Refreezing and Frozen Storage Effects on Muscle Functionality and Sensory Attributes of Frozen Cod (*Gadus morhua*). J. Food Sci..

[63] Hewitt M.R., Kreuzer R. (1969). Thawing of frozen fish in water. Freezing and Irradiation of Fish.

[64] Genç I.Y., Esteves E., Anibal J., Diler A. (2015). Effects of different thawing methods on the quality of meagre fillets. Ankara Üniversitesi Veteriner Fakültesi Dergisi.

[65] Kristinsson H.G., Kelleher S.D., Hultin H.O. (2009). Changes in Red Hake (*Urophycis chuss*) Muscle Induced by Different Freezing Strategies. J. Aquat. Food Prod. Technol..

[66] Schubring R. (2002). Double freezing of cod fillets: Influence on sensory, physical and chemical attributes of battered and breaded fillet portions. Food/Nahrung.

[67] Schubring R., Fikin K. (1998). Influence of double freezing on quality attributes of lean fish fillets during frozen storage as affected by rigor states. Advances in the Refrigeration Systems, Food Technologies and Cold Chain.

[68] Margeirsson B., Lauzon H.L., Pálsson H., Popov V., Gospavic R., Jónsson M.Þ., Sigurgísladóttir S., Arason S. (2012). Temperature fluctuations and quality deterioration of chilled cod (*Gadus morhua*) fillets packaged in different boxes stored on pallets under dynamic temperature conditions. Int. J. Refrig..

[69] Huss H.H. (1995). Quality and Quality Changes in Fresh Fish.

[70] Sivertsvik M., Jeksrud W.K., Rosnes J.T. (2002). A review of modified atmosphere packaging of fish and fishery products—Significance of microbial growth, activities and safety. Int. J. Food Sci. Technol..

[71] Kumar P., Ganguly S. (2014). Role of vacuum packaging in increasing shelf-life in fish processing technology. Asian J. Bio Sci..

[72] DeWitt C.A.M., Oliveira A.C. (2016). Modified Atmosphere Systems and Shelf Life Extension of Fish and Fishery Products. Foods.

[73] Tavares J., Martins A., Fidalgo L., Lima V., Amaral R., Pinto C., Silva A., Saraiva J. (2021). Fresh Fish Degradation and Advances in Preservation Using Physical Emerging Technologies. Foods.

[74] Corbo M.R., Altieri C., Bevilacqua A., Campaniello D., D’Amato D., Sinigaglia M. (2005). Estimating packaging atmosphere–temperature effects on the shelf life of cod fillets. Eur. Food Res. Technol..

[75] Sivertsvik M. (2007). The optimized modified atmosphere for packaging of pre-rigor filleted farmed cod (*Gadus morhua*) is 63 ml/100 ml oxygen and 37 ml/100 ml carbon dioxide. LWT.

[76] Wang T., Sveinsdóttir K., Magnússon H., Martinsdóttir E. (2007). Combined Application of Modified Atmosphere Packaging and Superchilled Storage to Extend the Shelf Life of Fresh Cod (*Gadus morhua*) Loins. J. Food Sci..

[77] Tsironi T.N., Taoukis P.S. (2018). Current Practice and Innovations in Fish Packaging. J. Aquat. Food Prod. Technol..

[78] Milijasevic J.B., Milijasevic M., Djordjevic V. (2019). Modified atmosphere packaging of fish—An impact on shelf life. Proceedings of the IOP Conference Series: Earth and Environmental Science, The 60th International Meat Industry Conference MEATCON2019.

[79] Roiha I.S., Tveit G.M., Backi C.J., Jónsson Á., Karlsdóttir M., Lunestad B.T. (2018). Effects of controlled thawing media temperatures on quality and safety of pre-rigor frozen Atlantic cod (*Gadus morhua*). LWT.

[80] Dalgaard P., Gram L., Huss H.H. (1993). Spoilage and shelf-life of cod fillets packed in vacuum or modified atmospheres. Int. J. Food Microbiol..

[81] Gram L., Huss H.H. (1996). Microbiological spoilage of fish and fish products. Int. J. Food Microbiol..

[82] Dalgaard P., Mejlholm O., Christiansen T., Huss H. (1997). Importance of Photobacterium phosphoreum in relation to spoilage of modified atmosphere-packed fish products. Lett. Appl. Microbiol..

[83] Hansen A., Mørkøre T., Rudi K., Olsen E., Eie T. (2007). Quality Changes during Refrigerated Storage of MA-Packaged Pre-*rigor* Fillets of Farmed Atlantic Cod (*Gadus morhua* L.) Using Traditional MAP, CO_2_ Emitter, and Vacuum. J. Food Sci..

[84] Dalgaard P., Hui Y.H. (2005). Microbiology of marine muscle foods. Handbook of Food Science, Technology, and Engineering.

[85] Hovda M.B., Sivertsvik M., Lunestad B.T., Rosnes J.T. (2007). Microflora Assessments Using PCR–Denaturing Gradient Gel Electrophoresis of Ozone-Treated and Modified-Atmosphere-Packaged Farmed Cod Fillets. J. Food Prot..

[86] Kuuliala L., Al Hage Y., Ioannidis A.-G., Sader M., Kerckhof F.-M., Vanderroost M., Boon N., De Baets B., De Meulenaer B., Ragaert P. (2018). Microbiological, chemical and sensory spoilage analysis of raw Atlantic cod (*Gadus morhua*) stored under modified atmospheres. Food Microbiol..

[87] Rotabakk B.T., Sivertsvik M. (2012). Solubility of carbon dioxide in muscle foods and its use to extend the shelf life of packaged products. Advances in Meat, Poultry and Seafood Packaging.

[88] Masniyom P. (2011). Deterioration and shelf-life extension of fish and fishery products by modified atmosphere packaging. Songklanakarin J. Sci. Technol..

[89] Lauzon H.L. (2010). Overview on fish quality research. Impact of fish handling, processing, storage and logistics on fish quality deterioration. Food Res. Innov. Saf..

[90] Aune T.F., Olsen R.L., Akse L., Ytterstad E., Esaiassen M. (2014). Influence of different cold storage temperatures during the Rigor mortis phase on fillet contraction and longer-term quality changes of Atlantic cod fillets. LWT.

[91] Ahmed I., Lin H., Zou L., Brody A.L., Li Z., Qazi I.M., Pavase T.R., Lv L. (2017). A comprehensive review on the application of active packaging technologies to muscle foods. Food Control.

[92] Kontominas M., Badeka A., Kosma I., Nathanailides C. (2021). Recent Developments in Seafood Packaging Technologies. Foods.

[93] Hansen A.Å., Moen B., Rødbotten M., Berget I., Pettersen M.K. (2016). Effect of vacuum or modified atmosphere packaging (MAP) in combination with a CO 2 emitter on quality parameters of cod loins (*Gadus morhua*). Food Packag. Shelf Life.

[94] Nielsen J., Hyldig G., Larsen E. (2002). ‘Eating Quality’ of Fish—A Review. J. Aquat. Food Prod. Technol..

[95] Roiha I.S., Jónsson Á., Backi C.J., Lunestad B.T., Karlsdóttir M.G. (2017). A comparative study of quality and safety of Atlantic cod (*Gadus morhua* ) fillets during cold storage, as affected by different thawing methods of pre-rigor frozen headed and gutted fish. J. Sci. Food Agric..

[96] Li Q., Zhang L., Luo Y. (2018). Changes in microbial communities and quality attributes of white muscle and dark muscle from common carp (*Cyprinus carpio*) during chilled and freeze-chilled storage. Food Microbiol..

[97] Yin X., Luo Y., Fan H., Wu H., Feng L. (2013). Effect of previous frozen storage on quality changes of grass carp (*Ctenopharyngodon idellus*) fillets during short-term chilled storage. Int. J. Food Sci. Technol..

[98] Jensen L.H.S., Nielsen J., Jørgensen B.M., Frosch S., Jã¸rgensen B.M. (2009). Cod and rainbow trout as freeze-chilled meal elements. J. Sci. Food Agric..

[99] Fagan J., Gormley T., Mhuircheartaigh M.U. (2004). Effect of modified atmosphere packaging with freeze-chilling on some quality parameters of raw whiting, mackerel and salmon portions. Innov. Food Sci. Emerg. Technol..

[100] Fagan J.D., Gormley T.R., Mhuircheartaigh M.U. (2003). Effect of freeze-chilling, in comparison with fresh, chilling and freezing, on some quality parameters of raw whiting, mackerel and salmon portions. LWT.

[101] Bøknæs N., Jensen K.N., Guldager H.S., Østerberg C., Nielsen J., Dalgaard P. (2002). Thawed Chilled Barents Sea Cod Fillets in Modified Atmosphere Packaging-Application of Multivariate Data Analysis to Select Key Parameters in Good Manufacturing Practice. LWT.

[102] Guldager H.S., Bøknæs N., Østerberg C., Nielsen J., Dalgaard P. (1998). Thawed Cod Fillets Spoil Less Rapidly Than Unfrozen Fillets When Stored under Modified Atmosphere at 2 °C. J. Food Prot..

[103] Vyncke W. (1983). Shelf life of thawed cod fillets kept in ice. Eur. Food Res. Technol..

